# Exercise prevents HFD-induced insulin resistance risk: involvement of TNF-α level regulated by vagus nerve-related anti-inflammatory pathway in the spleen

**DOI:** 10.1186/s13098-021-00712-w

**Published:** 2021-10-30

**Authors:** Zhengxi Huang, Jialing Tang, Kai Ji

**Affiliations:** 1 Department of Physical Education, Wuhan College, No 333, Huangjiahu Road, Wuhan, 430212 Hubei Province China; 2 Department of Physical Education, Central South University, Changsha, 410083 Hunan Province China; 3 College of Physical Education, Wuhan Sports University, Wuhan, 430212 Hubei Province China

**Keywords:** Exercise, Insulin resistance, Spleen, Tumor necrosis factor-alpha, High-fat diet

## Abstract

**Objectives:**

Regular physical exercise can improve insulin resistance in insulin target tissues. However, the mechanisms about the beneficial effect of exercise on insulin resistance are not yet fully resolved. This study was carried out to address whether insulin resistance improvement by exercise is involved in an anti-inflammatory pathway in the spleen in high-fat diet (HFD) feeding mice.

**Methods:**

Male C57Bl/6J mice with or without subdiaphragmatic vagotomy (sVNS) were subjected to medium-intensity treadmill exercise during HFD feeding. Glucose tolerance test and insulin tolerance test were detected, and spleen acetylcholine level, choline acetyltransferase activity (ChAT), protein kinase C (PKC) and tumor necrosis factor-alpha (TNF-α) were assayed.

**Results:**

We found that exercise significantly improves HFD-induced glucose intolerance and insulin resistance, along with an increase in acetylcholine level, ChAT activity, and PKC activity, and decrease in TNF-α level in the system and the spleen from HFD-fed mice. However, sVNS abolished the beneficial effect of exercise on glucose intolerance and insulin resistance, decreased acetylcholine level, ChAT activity, and PKC activity, and increase TNF-α level of the spleen in HFD-mice exercise intervention.

**Conclusions:**

These data reveal that the prevention of HFD-associated insulin resistance by exercise intervention involves reducing splenic TNF-α level, which is mediated by cholinergic anti-inflammatory activity via influencing PKC activity, ChAT activity, and acetylcholine concentration in mice spleen.

## Introduction

Insulin resistance is considered a pathological condition in which cells do not react efficiently to insulin stimulation. It is a decisive risk factor leading to diabetes, obesity, and cardiovascular disease, etc. The interplay of genetic and environmental factors results in insulin resistance, and the most critical environmental factors are high-fat dietary intake and physical inactivity [1]. In experiment studies, consumption of a high-fat diet (HFD) has been widely used to model insulin resistance, obesity, type 2 diabetes, etc., in rodents for investigating physiopathologic mechanisms of the diseases, therapy, and exercise benefits [2–4]. The pathogenesis of insulin resistance is complex, including insulin receptor mutation, endoplasmic reticulum stress, inflammation, oxidative stress, and mitochondrial dysfunction, which lead to damaged insulin signaling pathways and insulin resistance in some tissues, such as the liver, skeletal muscle, adipose tissue, and heart [1, 5]. In particular, chronic low-grade inflammation, which is reflected by elevated pro-inflammatory cytokines, has emerged as a central pathogenic mediator that can impair insulin signal transduction by affecting essential phosphorylation [1, 3]. Among cytokines, tumor necrosis factor-alpha (TNF-α) is verified to be the driver behind insulin resistance [6].

Numerous studies have demonstrated that regular physical exercise can improve insulin resistance in insulin target tissues, not dependent on body weight or fat mass reduction by exercise [1, 7]. The mechanisms underlying exercise to lessen insulin resistance are not yet fully resolved. Several pathways have been studied, such as autophagy activation [8], up-regulation of heat shock proteins expression [9], activation of insulin-independent glucose transport pathway, protecting against mitochondrial dysfunction [10], as well as reduction of low-grade inflammation [11]. It is reported that exercise also can enhance the vagal tone [12, 13]. That vagus nerve stimulation reduces pro-inflammatory cytokine production and improves insulin resistance in animal models with diet-induced obesity (DIO) [14]. Specifically, the vagus nerve can inhibit TNF-α production in the spleen, a crucial hub of peripheral anti-inflammation activity [15]. Splenectomy abrogates exercise control of serum TNF-α level in the context of endotoxemia [16]. However, it is unknown whether the beneficial effect of exercise on insulin resistance involves in TNF-α level regulated by vagus nerve-related anti-inflammatory activity in the spleen. This study was carried out to address whether splenic TNF-α level regulated by vagus nerve-related anti-inflammatory activity contributes to exercise-associated insulin resistance improvement in HFD mice. The results achieved will be helpful to the in-depth understanding of the exact mechanism of exercise protecting against insulin resistance and aid in providing novel sight of therapeutic intervention for insulin resistance-related disease.

## Materials and methods

### Animal and protocols

C57BL/6J male mice, 6–8 weeks old (Huafukang Co, Peking, China), were used in this study. The mice were maintained in groups of 5 under constant temperature (23 ± 2 °C), humidity (45–55 %), and 12-h light/dark cycle, with free access to water and food. Animal studies were approved by the Animal Utilization Committee of Wuhan College and conducted under NIH Guidance for the Care and Use of Laboratory Animals.

Mice received a high-fat diet with a diet of 5.26 kcal/g (HFD; 60 % kcal fat, Huafukang Co, Peking, China) or standard laboratory chow with 3.1 kcal/g (SD, 17 % kcal fat; Huafukang Co, Peking, China) as the control. A subset of mice fed with HFD was meanwhile subjected to moderate-intensity running treadmill exercise for 4 weeks. In addition, to observe the role of vague nerve-related anti-inflammatory pathways in exercise training, two groups of mice underwent subdiaphragmatic vagotomy (sVNS) or sham operation before the experiment. They were subjected to treadmill exercise during HFD feeding for 4 weeks. Therefore, there were 5 experimental groups: HFD, SD Control, HFD/exercise, HFD/sVNS/exercise, and HFD/sham/exercise (n = 8/each group). Mice that died, did not finish the exercise, or have data outliers have been excluded. After 4 weeks, all mice were subjected to intraperitoneal glucose tolerance tests and insulin tolerance tests. Then, a blood sample was taken from the retro-orbital sinuses into Eppendorf tubes containing EDTA, and plasma was prepared and frozen at − 80 °C. Subsequently, mice were anesthetized via intraperitoneal injection of sodium pentobarbital (1 %, 50 mg/kg), and a midline laparotomy was performed. Spleen was dissected and put immediately in tubes on dry ice and stored at − 80 °C until analysis.

### Subdiaphragmatic vagotomy (sVNS)

The surgery was performed following the method described by Torres-Rosas et al. [17]. In short, after anesthetizing the mice with sodium pentobarbital (1 %, 50 mg/kg), incise the abdomen and gently push open the liver and stomach to expose the esophagus. Then, the ventral and dorsal branches of the vagus nerve were separated under an operating microscope, and the nerve about 3 mm in length was excised. For the sham operations, the surgical procedures were identical but without transection of the vagal nerve.

### Treadmill exercise

Treadmill running exercise was initiated at the onset of an HFD. After 3 days of training at 10–15 m/min for 10 min once before the experiment, mice in all exercise groups performed treadmill running using a treadmill for rodents (MK-680; Muromachi, Tokyo, Japan) 30 min/day, 5 days/week, for 4 consecutive weeks. Each session is always in the afternoon. Each mouse runs at a different time each exercise day. Each exercise began with a 10-minute warm-up at 6 m/min and was then followed by 8 m/min and gradually increases to 18 m/min on the treadmill at 0° incline. Mice that stopped running were gently pushed back onto the belt. This intensity exercise is considered as medium-intensity treadmill exercise, corresponding to 65–70% of VO_2max_ [18], which is the most common exercise recommended to improve insulin resistance and lipid profile [19].

### Glucose and insulin tolerance tests

Glucose tolerance was assessed by intraperitoneal glucose tolerance test (IPGTT) after 12 h of starvation (21:00–9:00). Mice were injected with 50 % D-Glucose solution intraperitoneally at a dose of 2 g/kg body weight, and blood samples were obtained from the tail after 0, 30, 60, 90, and 120 min. Blood glucose was measured by a One Touch Ultra-portable glucometer (Elite; Bayer Inc.).

Insulin sensitivity was assessed by an insulin tolerance test (ITT). Given that insulin rapidly lowers blood glucose levels, mice have fasted for only 6 h (7:00–13:00) before ITT. After weighing the mice, mice were injected with insulin (0.75 IU/kg body weight, Novo Nordisk, Denmark) intraperitoneally. The blood sample was taken from the tail at 0, 30, 60, 90, and 120 min, and blood glucose was measured.

### Biochemical analyses

The spleen samples were homogenized in ice-cold sodium phosphate buffer (pH 7.4) with a protease inhibitor. Homogenates were centrifuged at 10,000 × *g* for 5 min at 4 °C, and supernatants were collected. Acetylcholine concentrations, choline acetyltransferase (ChAT) activity were measured using ACH/ChAT assay kit (MLBIO Biotechnology Co. Shanghai, China), and protein kinase C (PKC) activity was detected by ELISA using PKC activity assay kit (Enzo Biochem, NY, USA), according to the manufacturer’s protocol. The levels of TNF-α were analyzed using a commercially available enzyme-linked immunosorbent assay kit (Lianke, Hangzhou, China) following the manufacturer’s instructions. Protein concentrations were quantified using a bicinchoninic acid protein assay kit (MLBIO Biotechnology Co. Shanghai, China).

### Statistical analysis

Data were expressed as mean ± SD. The data were analyzed by one-way analysis of variance (ANOVA), followed by Tukey test for multiple groups, or Student’s t-tests of unpaired samples for the statistical difference between groups, using The GraphPad Prism 6.0 software. P < 0.05 was regarded as a statistically significant difference.

## Results

### Exercise ameliorates HFD-induced insulin resistance in male mice

**Fig. 1:**
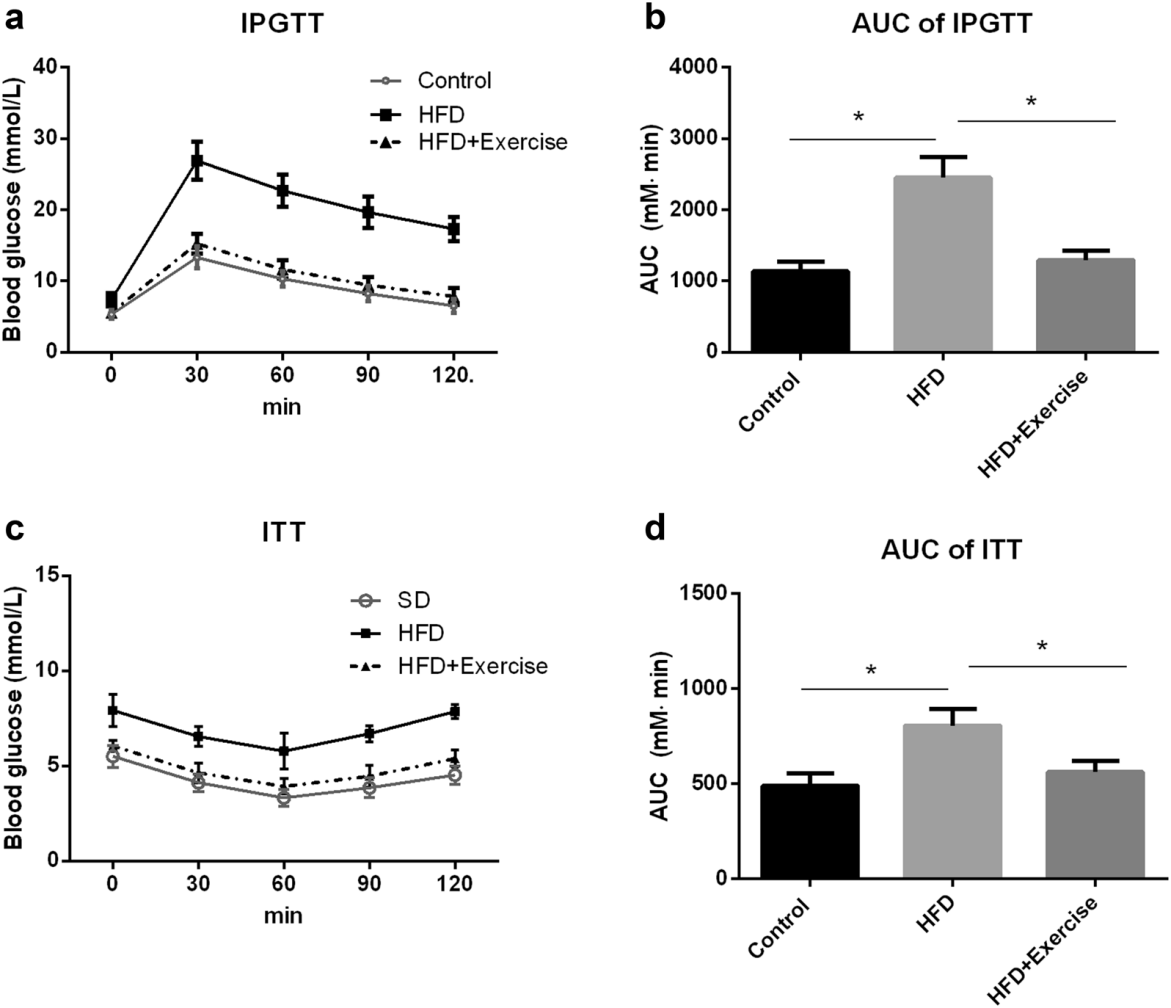
Effect of exercise on glucose tolerance and insulin sensitivity in HFD-fed mice. Male C57Bl/6J mice were subjected to treadmill exercise or no exercise during HFD feeding. After 4 weeks, the intraperitoneal glucose tolerance test (IPGTT) and insulin tolerance test (ITT) were utilized to evaluate glucose tolerance and insulin sensitivity, respectively. To depict significant changes between groups, the area under the curve (AUC) of IPGTT and ITT was calculated by the trapezoid method. **A** IPGTT curve. **B** the AUC of IPGTT. **C** ITT curve. **D** the AUC of ITT. Values are mean ± SD. n = 8 each group, * p < 0.05

Male C57Bl/6J mice were subjected to medium-intensity treadmill exercise or no exercise during HFD feeding for 4 weeks. IPGTT and ITT were utilized to evaluate glucose tolerance and insulin sensitivity. To depict significant changes between groups, the area under the curve (AUC) of IPGTT and ITT was calculated by the trapezoid method. Consist with other studies [2, 20], HFD-feeding mice have lower glucose tolerance (Fig. 1A), and the AUC of IPGTT significantly increased compared to standard chow-fed control mice (Fig. 1B). However, HFD-feeding mice with exercise showed a notable improvement of glucose tolerance (Fig. 1A), and its AUC significantly decreased compared to mice without exercise (*p* < 0.05) (Fig. 1B). Similar results were also observed in ITT. Insulin resistance occurred in HFD-feeding mice but was improved in HFD-feeding mice with exercise (Fig. 1C), and the AUC of ITT showed a significant difference between exercise and no exercise mice (*p* < 0.05) (Fig. 1D). These results indicate that exercise prevents HFD-induced glucose intolerance and insulin resistance in male mice.

### Exercise reduces TNF-α level and increases the acetylcholine level in the spleen

**Fig. 2:**
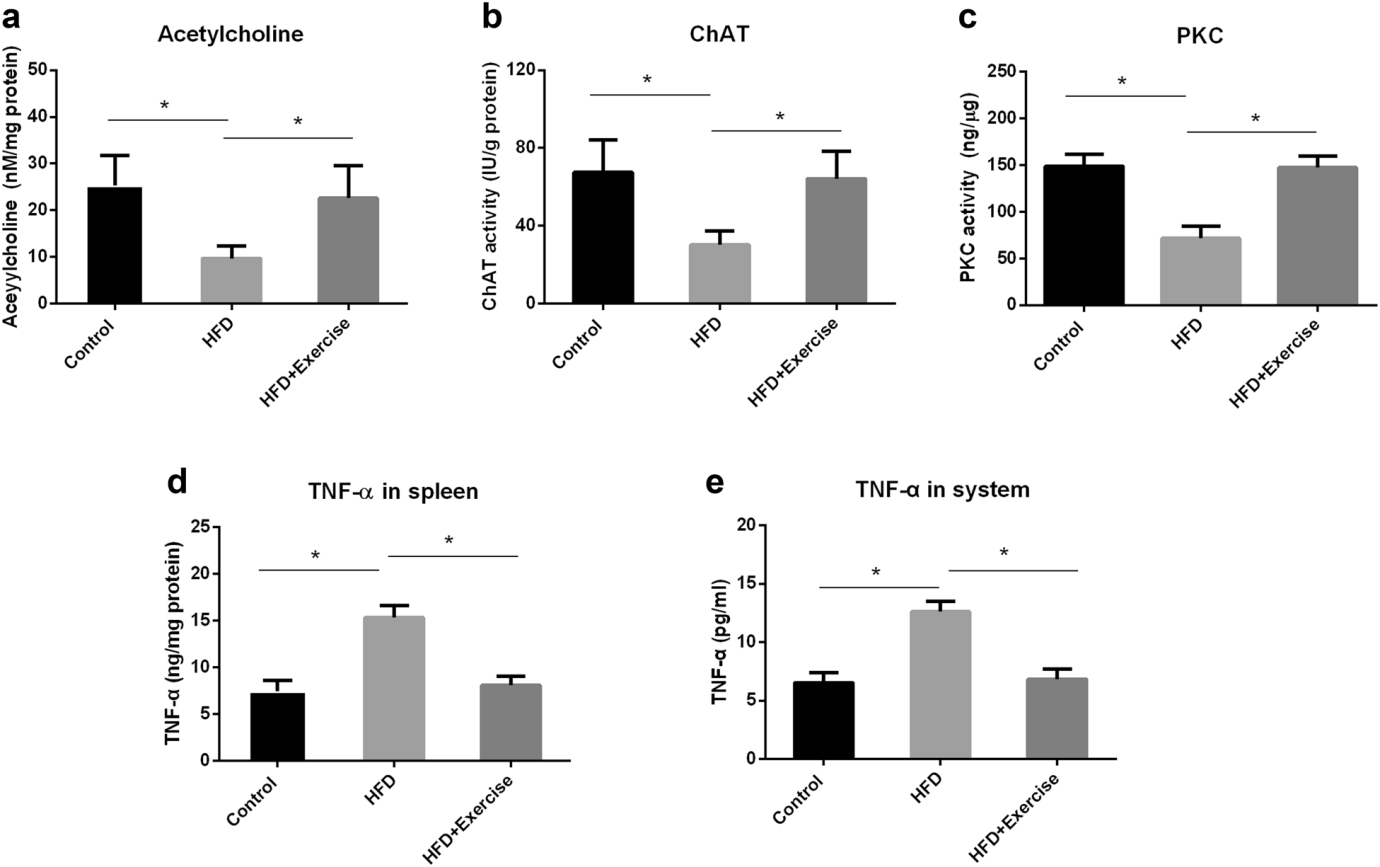
Exercise increases anti-inflammatory activity in the spleen and reduces systemic TNF-α levels in HFD-fed mice. Male C57Bl/6J mice were subjected to medium-intensity treadmill exercise or no exercise during HFD feeding. After glucose and insulin tolerance tests, blood samples and the spleen tissue were taken. Acetylcholine level, choline acetyltransferase activity (ChAT), protein kinase C (PKC) activity, and tumor necrosis factor-α (TNF-α) level were analyzed. **A** Acetylcholine level in the spleen. **B** ChAT activity in the spleen. **C** PKC activity in the spleen. **D** TNF-α level in the spleen. **E** TNF-α level in the system. Values are mean ± SD. n = 8 each group, * p < 0.05

According to reports, in the spleen, choline acetyltransferase ^+^ T cells (ChAT ^+^ T cells) can synthesize and release acetylcholine in response to outgoing vagus nerve signals. Acetylcholine then activates the nicotinic acetylcholine receptor α7 subunit (α7nAChR) on macrophages to inhibit the production and release of cytokines through macrophages [21]. ChAT catalyzes the biosynthesis of acetylcholine, which plays a vital role in regulating anti-inflammatory activity [21]. The protein kinase C (PKC) pathway has been shown to mediate the up-regulation of ChAT transcription and acetylcholine release and regulate cholinergic activity [22]. To study the effect of exercise on the cholinergic activity of the spleen, we next measured the acetylcholine level, ChAT activity, PKC activity, and TNF-α level in the spleen of sacrificed mice. We found that HFD consumption effectively reduces acetylcholine level (*p* < 0.05) (Fig. 2A), ChAT activity (*p* < 0.05) (Fig. 2B), and PKC activity (*p* < 0.05) (Fig. 2C), and simultaneously increases TNF-α level compared to standard chow-fed control (*p* < 0.05) (Fig. 2D). By contrast, mice with exercise have a significant increase in acetylcholine level (*p* < 0.05) (Fig. 2A), ChAT activity (*p* < 0.05) (Fig. 2B) and PKC activity (*p* < 0.05) (Fig. 2C), and a decrease in TNF-α level compared to mice without exercise (*p* < 0.05) (Fig. 2D).

Furthermore, we also detected systemic TNF-α levels in HFD-fed mice with or without exercise. Consistent with other studies [3], HFD consumption significantly elevated systemic TNF-α levels compared to standard chow-fed control (*p* < 0.05) (Fig. 2E). But, exercise prevented the increase in systemic TNF-α levels in HFD-fed mice. These results suggested that exercise increased splenic anti-inflammatory activity and attenuated TNF-α levels in the system and the spleen.

### sVNS nullifies the potential of exercise to improve insulin resistance in HFD-fed mice

**Fig. 3:**
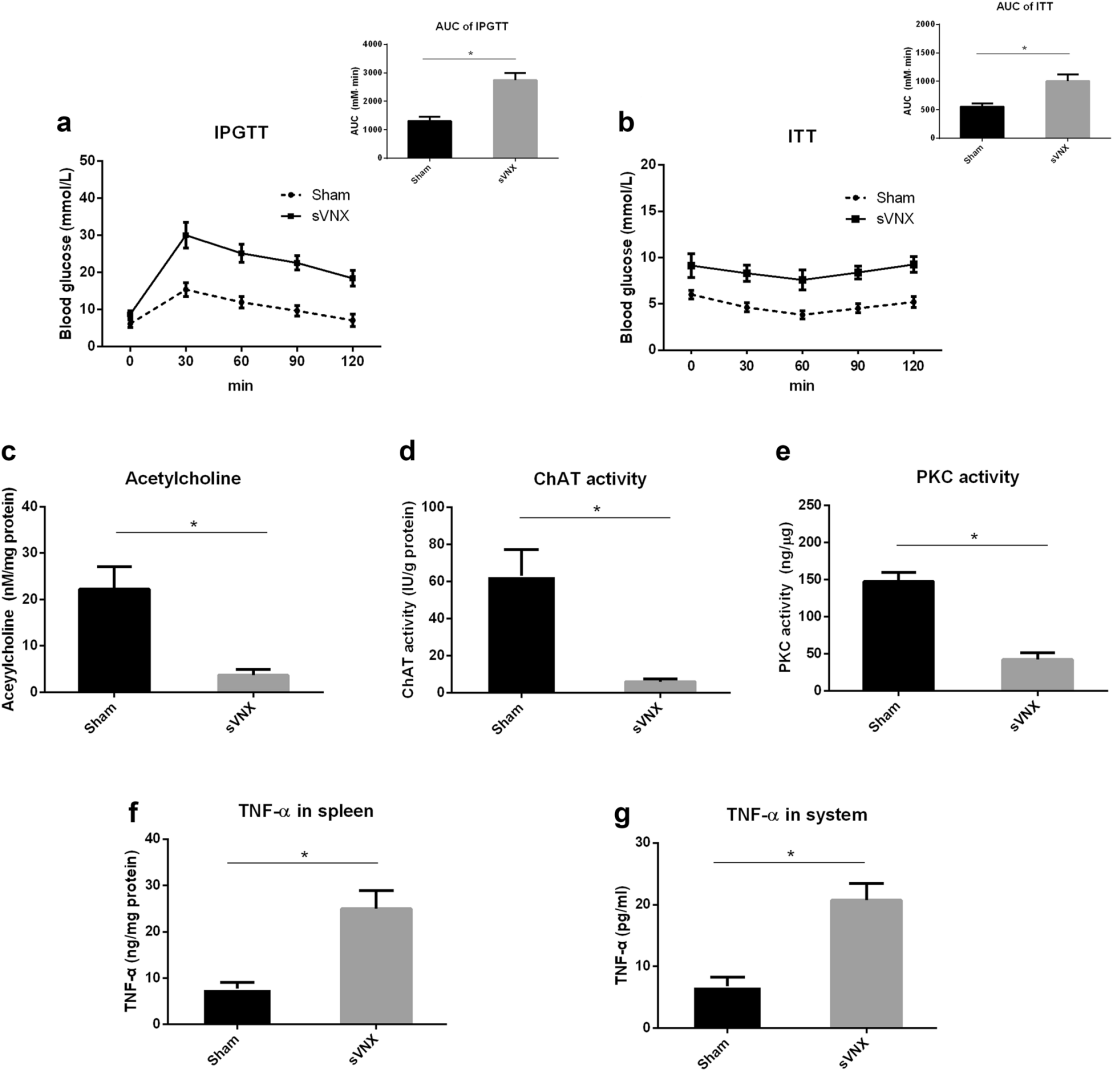
sVNS abrogates the beneficial effect of exercise in HFD-fed mice. Mice with or without sVNS were subjected to treadmill exercise during HFD feeding. After 4 weeks, intraperitoneal glucose tolerance test (IPGTT) and insulin tolerance test (ITT) were utilized to evaluate glucose tolerance and insulin sensitivity, respectively, and the area under the curve (AUC) of IPGTT and ITT was calculated by the trapezoid method. Then blood samples and the spleen tissue were taken for analysis. **A** IPGTT curve and its AUC. **B** ITT curve and its AUC. **C** Acetylcholine level in the spleen. **D** ChAT activity in the spleen. **E** PKC activity in the spleen. **F** TNF-α level in the spleen. **G** TNF-α level in the system. Values are mean ± SD. n = 8 each group, * p < 0.05

To further study whether the improvement of exercise-induced insulin resistance is mediated by the vagus nerve-related anti-inflammatory activity in the spleen, the mice underwent sVNS surgery before the experiment. The subphrenic vagus nerve has been shown to mediate the cholinergic anti-inflammatory activity of the vagus nerve and control the production of TNF-α in the spleen [23]. Then, sVNS mice were subjected to treadmill exercise during HFD feeding. After 4 weeks, sVNS mice did not display improvement of glucose tolerance by exercise and had a higher AUC of IPGGT than that of sham operation mice with exercise (*p* < 0.05) (Fig. 3A). Similar results were also observed in ITT. Insulin resistance was not improved by exercise, and its AUC is higher in surgery mice than that in sham mice (*p* < 0.05) (Fig. 3B). At the same time, sVNS caused a significant decrease in the acetylcholine level (*p* < 0.05) (Fig. 3C), ChAT activity (*p* < 0.05) (Fig. 3D) and PKC activity (*p* < 0.05) (Fig. 3E) in the spleen. The TNF-α level in the spleen (*p* < 0.05) (Fig. 3F) and in the system increased (*p* < 0.05) (Fig. 3G) in HFD mice with exercise. All in all, these results indicate that exercise can prevent HFD-induced insulin resistance by increasing the anti-inflammatory activity related to the vagus nerve in the spleen.

## Discussion

This study found that treadmill running training reduced TNF-α levels in the system and the spleen and increased acetylcholine concentration and ChAT activity related to the vagus nerve-based splenic anti-inflammatory activity, along with improved glucose intolerance and Insulin resistance in HFD mice. We also demonstrated that subdiaphragmatic vagus nerve amputation eliminated the beneficial effects of exercise training on splenic TNF-α levels, acetylcholine concentration, and ChAT activity and prevented exercise from improving insulin resistance in HFD mice. It shows that the level of TNF-α is affected by the vagus nerve to regulate the nerve-related anti-inflammatory activity in the spleen, which can help improve glucose intolerance and insulin resistance through exercise.

HFD-fed C57BL/6J mouse model is an essential and reliable model for investigating impaired glucose tolerance, insulin resistance, type 2 diabetes mellitus, their new treatments, as well as exercise benefits [2, 3]. Different durations for HFD intervention show the further extent of the effect on some parameters [3]. After 1 week of HFD consumption, circulating glucose increased, glucose tolerance and insulin sensitivity were impaired, and insulin resistance occurs in many tissues, specifically, the skeletal muscle, liver, fatty tissue, and heart [2, 24]. Heydemann proposes that the feeding period is from 4 to 20 weeks [3]. In the present study, the HFD intervention period is 4 weeks, in which glucose intolerance and insulin resistance were observed. Moreover, given that male mice were susceptible enough to HFD-related chronic inflammation [25], we chose male mice in the present study.

Vagus nerve regulation of peripheral anti-inflammatory activity is mediated via innervation of some organs or tissues, including the liver, gastrointestinal tract, and spleen [26]. Specifically, the spleen is a crucial hub of vagus nerve regulation of anti-inflammation [16, 27]. It is shown that efferent vagal stimulation can recruit nonneuronal ChAT^+^ T cells to the spleen [27]. Nonneuronal ChAT^+^ T cells can relay vague nerve signals to synthesize and release acetylcholine that regulates cytokine release in the spleen [20]. In ChAT^+^ T cells, ChAT catalyzes acetylcholine biosynthesis from choline and acetyl-coenzyme A [21]. It has shown that PKC activation mediates up-regulation of ChAT mRNA expression and acetylcholine release through PKC-mitogen-activated protein kinase (MAPK) signal pathway in human and rodent T lymphocytes [22]. ChAT^+^ T cells have emerged as essential for the regulation of low-grade systemic inflammation [20]. Vagus nerve stimulation increases the concentration of acetylcholine in the spleen, inhibits the production of TNF-α by macrophages, and reduces serum TNF-α levels during endotoxemia [19]. In this study, we found that moderate-intensity exercise can also increase the acetylcholine concentration, ChAT activity, and PKC activity in the spleen and reduce the level of TNF-α in the spleen and the system in HFD-fed mice. These findings prove that exercise enhances PKC activity and subsequently ChAT activity and acetylcholine concentration. In the spleen, the elevated acetylcholine, as intermediary messengers, acted on α7nAChR to suppress the synthesis and release of TNF-α by macrophages [28]. In vitro, antigen-stimulated spleen cells from α7nAChR-deficient mice can release significantly more significant amounts of TNF-α [28]. Moreover, it appears that vagus nerve regulation of anti-inflammation in the spleen is not confined to effects on ChAT^+^ T cells and macrophages. Vagus nerve output also regulates mobilization and antibody secretion of B-cells and pro-inflammatory cytokine production by CD4^+^ T cells [29].

However, there is no compelling evidence that the vagus nerve innervates the spleen directly. It is proposed that the spleen-projecting efferent vagus nerve (one of the subdiaphragmatic vagus nerve branches) terminates in the celiac-superior mesenteric plexus ganglion, from which catecholaminergic fibers innervates the spleen [30]. Several studies indicated that subdiaphragmatic vagus nerve stimulation or interruption could affect anti-inflammatory activity in the spleen from rodents [21, 22]. In the current study, we performed sVNS to evaluate the role of splenic cholinergic activity in insulin resistance improvement by exercise. We found that sVNS abolished exercise control of systemic and splenic TNF levels.

In the present study, we detected TNF-*α*, an important pro-inflammatory cytokine, because TNF-*α* is used as a marker for the dynamic change of low-grade inflammatory responses to exercise and high-fat meals [31] and involves insulin resistance [6]. In addition, it is also a significant cytokine affected by vagus nerve-related anti-inflammatory activity [27]. Studies show that TNF-α incubation inhibits insulin-stimulated glucose uptake and leads to insulin resistance in adipocyte cell lines [1]. However, antibody neutralization of TNF-α and gene knockout of TNF-α and its receptors improve insulin sensitivity and increase glucose uptake in response to insulin in diet-induced obese mice [32, 33]. Multiple mechanisms have been proposed to account for the effect of TNF-α on insulin resistance, including altering the phosphorylation status of the insulin-signaling molecules, triggering the production of other inflammatory factors, down-regulating the expression of insulin action-related genes, etc. [1, 34].

It is well known that regular exercise can restrain chronic inflammation markedly in experimental animals and humans [35, 36]. In the HFD-fed mouse obese model, obvious increases of pro-inflammatory cytokines were observed in some tissues and circulation, and exercise training prevents this chronic inflammation [36]. The mechanism underlying the exercise effect seems far more than energy consumption [36]. In recent years, studies show that the mechanism involves multiple pathways, such as adaptive epigenetic modifications [37], the interplay between skeletal muscle and immunity tissues [38], the suppression of inflammatory signaling pathways [39]. An increase in vagal tone also has been verified to contribute to exercise control of chronic inflammation [12]. It is reported that regular physical exercise increases central 5-hydroxytryptamine synthesis, which subsequently promotes vagal modulation in rats [12]. In the present, we demonstrate that increased acetylcholine concentration and ChAT activity in the spleen contributes to TNF-α reduction by exercise.

The effectiveness of exercise usually depends on the modality, intensity, frequency, and duration of exercise. Previously published studies used different protocols to improve insulin resistance and achieved successful outcome in HFD-fed mouse model. For example, Wang et al. [40] reported that a treadmill exercise with a VO2max intensity of 75–90% was performed for 60 min a day for 4 weeks, 6 days a week; Pereira reported a training program that uses power climbing training weight-bearing equipment, 1 time a day, 5 days a week, a total of 15 times [41]; the other two studies used medium-intensity and high-intensity treadmills, 40 min each time, 3–5 times a week for 8 to 10 weeks, and the results showed that high Intensity training is more effective than moderate intensity training in improving insulin resistance [42, 43]. In the present study, we chose a medium-intensity treadmill exercise for 30 min/day, 5 days/week, for 4 weeks, which markedly prevents insulin resistance.

## Conclusions

To sum up, our data show that the prevention of HFD-related insulin resistance by exercise intervention involves the decrease of TNF-α level in the spleen, which is mediated by cholinergic anti-inflammatory activity by affecting PKC activity, ChAT activity and acetylcholine concentration in mouse spleen. The results achieved will add to our understanding of the mechanism of exercise to improve insulin resistance and aid in providing novel sight of therapeutic intervention for insulin resistance-related disease.

The animal model used in the experiment was approved by the Academic Ethics Committee of Wuhan College.

## Data Availability

All the original data of the article and the archived files of the experiment process could be obtained from the corresponding author with permission.
